# Memorial Service to Dr. W. F. Holt

**Published:** 1901-11

**Authors:** K. P. Moore, H. McHatton, H. J. Williams, M. A. Clark, C. H. Hall

**Affiliations:** Committee; Committee; Committee; Committee; Committee


### MEMORIAL SERVICE TO DR. W. F. HOLT.

The Macon Medical Society met last night and held a memorial service for Dr. William F. Holt. The following resolutions were adopted:

The Macon Medical Association has the sad office of paying its late tribute to its oldest and most honored member, Dr. William Flewellen Holt.

Dr. Holt was the son of a physician, whose early death was the source of greatest sorrow to Macon. Dr. Abner Flewellen Holt was one of the leading men in his profession and was greatly beloved by the people of this city. His son resolved early to take his father's vacated place, and after graduating in medicine in the same college from which his father received his diploma, the Jefferson Medical College of Philadelphia, he opened an office in Macon. He forged his own way. He had no patron nor helper. Well equipped, he worked on and waited patiently for success to come. He won his place in the front rank. He was a man of clear head and fine attainments, and being relieved from the pressure of narrow circumstances he was able to secure for himself the best aids. He read the best books and journals, and as often as possible made it a rule to visit the best hospitals in the larger cities and study all new methods of treatment. His merits were recognized by his brethren of the faculty and they paid him all honor where it was possible.

He was chairman of the local association and president of the State association. He began the practice in the days of Hammond, Boone, Fitzgerald, Nottingham, Lightfoot, Mettauer, Harrison and Green, a faculty famous for its ability and high tone, and outlived all his early colleagues, save our venerated Mettauer. He had no art for gaining popular favor. He was above all pretense, all trickery, and knew nothing of professional envy or jealousy. He was particularly kind to young men who never felt that he considered himself as their superior. To his equals he was always courteous and considerate, and to his inferiors he was more than kind. He was devoted to his work, he did nothing by starts. During his office hours he was always in his office, and the rest of his time, except when at home after tea, he was in his carriage visiting his patients. Many of them were poor and could render him no return for the full service he gave them. He was deeply sympathetic and untiring. He never said a rude thing nor did a rude act. He was a gentleman of the highest type, incapable of doing a dishonorable act, and his brethren considered him as the incarnation of the best ethics of the profession. Do unto others as you would they should do unto you.

Your committee beg leave to submit the following resolutions:

*Resolved*, That this association recognizes an irreparable loss in the death of Dr. William Flewellen Holt.

*Resolved*, That his high tone, dignified courtesy and beautiful character commend him to the whole profession as one of its worthiest members whose example is deserving of closest imitation.

*Resolved*, That we tender to Miss Ida Holt and her brother, Mr. William Holt, our very kindest sympathy and assure them of our warmest regard.

*Resolved*, That this paper be published in the MEDICAL JOURNAL and in our city papers.

K. P. MOORE,
H. MCHATTON,
H. J. WILLIAMS,
M. A. CLARK,
C. H. HALL,
*Committee.*

### DR. HALL'S ADDRESS.

Dr. C. H. Hall made the following address:

It is well to lay down our work when we have reached the zenith—in the fullness of our mental powers—before any evidence of senility is apparent—to die as I heard our deceased friend say, "with the harness on." Our profession is a lottery and requires something beyond knowledge for its success. Like a poet, the successful doctor is "*nascitur non fit.*" Holt had in the sick-room this indefinable something. His patients felt it. He created a medical atmosphere around him. Coupling with this his collegiate and medical equipment, we have the keynote of his success. It was his lot not to be nurtured in the school of poverty, self-denial and hardship, but it did not spoil him, and he was none the less able to sympathize with those who had these trials and difficulties. Such a man lived not in vain. He has shown us in his own person how the pilgrimage of life can manfully be gone through. I first knew him in 1863. He was singularly handsome and attractive in person and manner. Hospitable and sociable in his feelings, he was never happier than in the company of those he esteemed. As a host he was unexcelled, and he knew just how to make each and every guest feel that he or she was the one he delighted to honor. I shall miss him in his home. He loved the company of the young and light-hearted, and his gentle courtesy and ready sympathy won for him a warm place in their hearts. He died before age had loosed the silver cord which bound him in kindly interest to those around him.

It would be a grateful theme to dwell on his personal virtues and his professional merits, but why should I? You all knew them well, but none so well perhaps as his friend of more than thirty years. To his profession he was enthusiastically attached. It was to him not a mere business to gratify his individual and social necessities, but a noble ministry, feeling with Cicero, that "man approaches to the gods in nothing so much as giving health to man." Holt, imbued with the sentiment "that to preserve and restore is little less than to make," pursued his profession as a philanthropist. Nobly he performed his duty to himself, to society, to his God. Peace be to him!

